# ‘I am not very well I feel nearly mad when I think of you’: Male Jealousy, Murder and Broadmoor in Late-Victorian Britain

**DOI:** 10.1093/shm/hkw045

**Published:** 2016-05-07

**Authors:** Jade Shepherd

**Keywords:** asylums, Broadmoor, emotions, insanity, jealousy

## Abstract

This article compares the representations of jealousy in popular culture, medical and legal literature, and in the trials and diagnoses of men who murdered or attempted to murder their wives or sweethearts before being found insane and committed into Broadmoor Criminal Lunatic Asylum between 1864 and 1900. It is shown that jealousy was entrenched in Victorian culture, but marginalised in medical and legal discourse and in the courtroom until the end of the period, and was seemingly cast aside at Broadmoor. As well as providing a detailed examination of varied representations of male jealousy in late-Victorian Britain, the article contributes to understandings of the emotional lives of the working-class, and the causes and representations of working-class male madness.

The evening of 21 December 1884 began like any other for 17-year-old Laura Wilson; she played and sang hymns with her sister at her parent’s home in Woolwich. At 10.00 pm she returned next door to the home of Sarah Hewitt where she worked as a servant. Early the next morning, Hewitt was in her bedroom when she heard a scuffle. Moments later, a distressed Laura staggered in and cried, ‘I am stabbed, I am stabbed to the heart’ before fainting.^1^ Awoken by Hewitt banging on the wall to rouse them, Laura’s father, William, reportedly turned to his wife and said, ‘I believe there is something wrong with Laura and that Fred.’ They rushed next door where, as William later recalled, ‘[I] saw my poor girl lying on the floor motionless … she groaned slightly, but all signs of life soon disappeared.’^2^ Fred was 21-one-year-old Frederick Marshall, who had previously been Laura’s betrothed. Soon after Laura began working for Hewitt, Marshall reportedly became jealous of her relationship with another man, and Laura subsequently called off the engagement.^3^ This reportedly exacerbated Marshall’s jealousy, resulting in Laura’s murder and Marshall’s committal into Broadmoor Criminal Lunatic Asylum.

Broadmoor, which opened in 1863, was the first asylum of its kind in England and Wales.^4^ It housed Queen’s pleasure patients, who had been found insane before or during their trial, and insane convicts, who had developed insanity in prison. The cases of men who murdered or attempted to murder their wives or sweethearts before being found insane and committed into Broadmoor between 1864 and 1900 are the foundation of this article. They underpin an examination of male jealousy in late-Victorian Britain, which compares the representations of jealousy in popular culture and medical literature, and in these men’s trials and diagnoses at Broadmoor. This article contributes to scholarship on the causes and representations of working-class male madness.^5^

There is much scholarship detailing the histories of individual emotions, but little on historical representations of jealousy in Britain.^6^ Martin Wiener and Carolyn Conley have examined the acceptance of jealousy as a defence in the British courtroom, Valerie Pedlar and Stephen Kern have examined jealousy in British fiction, and Ronald Rae Mowat studied *Morbid Jealousy and Murder* in Broadmoor in post-war Britain.^7^ Scholarly work on the emotions has tended to privilege cultural and intellectual history approaches.^8^ As Claire Langhamer noted, the field ‘has often looked more like history from above than from below’.^9^ Historians have begun ‘to move beyond social prescription and to examine lived experience’, and this article contributes to such efforts to retrieve the emotional lives of the non-elite.^10^ All of the cases examined here are those of working-class men, and some reveal patients’ subjective accounts of their emotions.^11^ Working-class voices—those of patients’ friends and families—appear in trial proceedings, press reports and letters contained in asylum case files.

The cases were identified using Broadmoor’s admission registers for the years 1864–1900.^12^ I examined the cases of all of the 45 men who were admitted having been found insane after murdering or attempting to murder their wives or sweethearts, for whom case files were accessible. These case files are selective records of patients’ asylum lives, but contain medical reports, memorandums written by asylum staff, and letters to and from patients, patients’ relatives and friends, and the authorities. Despite the selectivity of medical records, Jonathan Andrews has highlighted their potential for examining ‘medical discourse and ideologies’.^13^ Here, they enable examination of the position of jealousy at Broadmoor.

Much scholarship has detailed the crimes and trials of individuals who were committed to Broadmoor, but the opening of the Broadmoor archive allows us to follow patients from their crime through their trial and into the asylum.^14^ Here, once cases were identified, patients’ crimes and trials were researched using newspaper articles and *Old Bailey Trial Proceedings Online* (OBPO), following Joel Eigen and Martin Wiener, amongst others, who have used OBPO to reconstruct trials and to gauge lay, medical and legal opinion.^15^ While trial transcripts are invaluable for this task, they are not complete verbatim accounts and legal arguments were sometimes omitted.^16^ To create the most comprehensive accounts possible, trial reports in *The Times* and the local press have been consulted. Press reports of assizes court proceedings were also crucial, as not all of the cases were tried at the Old Bailey. Newspaper representations have the imperfection of being filtered through a middle-class lens, but in a number of instances the verbatim publication of letters written by defendants before or during trials provides an unusual opportunity to hear their ‘voice’. Tracing the representations of these men through their crimes, trials and into Broadmoor allows close attention to be paid to those cases where jealousy was considered a factor, and combining this with an examination of jealousy in popular literature, press reports, medical literature and legal works, reveals much more about how jealousy was conceived by the medical profession and how the passion was represented in wider society than can be established from archival records alone. Comparing representations of male jealousy within and across these social, cultural, medical and legal discourses provides a fresh and comprehensive insight into how the passion was perceived in the late-Victorian period. This article sits alongside work on subjective accounts of mental illness, whilst also contributing to histories of the emotions, medicine, asylums and criminal lunacy.^17^

## Jealousy in Literature and the Press

By the mid-nineteenth century, jealousy, a powerful theme since the Greek classics, had long been condemned.^18^ Seventeenth-century Jesuits characterised jealousy as a ‘monstrous’ passion and William Shakespeare described jealousy’s ‘misery’ and ‘venom’ in *Othello*.^19^ Nineteenth-century novels, serialised fiction and the press all depicted the harrowing effects jealousy could have on individuals and their loved ones.^20^ Literary scholars have established that nineteenth-century authors of fiction engaged with various medical debates.^21^ Anthony Trollope’s novel *He Knew He Was Right* (1869) was, a critic in the *British Quarterly Review* observed, a ‘psychological’ study of the ‘the rise and development of jealous monomania’.^22^ Here, such sources are used to understand how jealousy was represented to the nineteenth-century reading public.

Peter Stearns writes that for some nineteenth-century Americans ‘jealous possessiveness and love went hand in hand’.^23^ In Britain, it was more commonly believed that ‘an ideal affection will be entirely free from jealousy’.^24^ Journalists and authors of fiction overwhelmingly regarded jealousy as a ‘bad passion’ that was ‘absurd’ and ‘unreasonable’.^25^ The *Saturday Review* warned readers about the ‘nightmare’ that was jealousy, its hazardous effects including madness.^26^ British fiction also depicted true love as jealousy free. In *He Knew He Was Right* the narrator states that protagonist Mr Trevelyan, who was driven to madness through his jealousy and suspicion of his wife’s infidelity with Colonel Osbourne, must have been mad or evil ‘or he could not have tortured as he had done, the woman whom he owed the closest protection which any one human being can give to another’.^27^

Representations of jealous men in literature were couched in the language of savagery and violence: they were wild, untrusting ‘green-eyed monsters’ who lacked self-control and rationality. In her *Jealousy and Revenge Tales* (1845) Eliza Peakes described protagonist Arthur Courtwright’s ‘bursts of frantic passion’ and illustrated ‘the hideous monster’ jealousy made him.^28^ In the serialised story ‘No, Never!’ (1885) overt references to *Othello* were used to tell the tale of Dougauld McGregor who was courting 18-year-old Mona.^29^ References are made to ‘[t]he green-eyed monster of jealousy … gnawing away at [Dougauld’s] heart’, to his ‘violent agitation of mind’, ‘fiercely rolling eyes’ and threats of violence.^30^ In the short story ‘Caleb’s Jealousy’ (1876), Caleb Moor, whose surname readers could not have failed to link with Othello, the Moor of Venice, feared his betrothed loved another man. Jealousy made the previously kind Caleb a ‘madman’, ‘a revengeful, dark-browed sort of fellow, with all sorts of wicked thoughts and feelings’ who grabbed a rifle intending to murder.^31^

An examination of Victorian literature highlights two distinct causes of jealousy. First, jealousy caused by the violation of feelings that occurred because of infidelity or unrequited love.^32^ In 1871 eminent Victorian minister R. W. Dale defined jealousy as ‘the anger and pain of injured and insulted love’.^33^ According to some commentators, this destroyed or injured love ‘awakens Combativeness to resent the injury, and in some cases Executiveness or Destructiveness, to revenge it’.^34^ The second cause of jealousy was a perceived violation of property: men believed that their wives or sweethearts belonged solely to them and the fear that someone would steal that property caused their jealousy. Some journalists, judges, juries and social commentators associated jealousy and possession with domestic abuse and the working classes.^35^ Social reformer Frances Power Cobbe asked:

How does it come to pass that while the better sort of Englishmen are thus exceptionally humane and considerate to women, the men of the lower class of the same nation are proverbial for their unparalleled brutality, till wife-beating, wife-torture, and wife-murder have become the opprobrium of the land?^36^

Henry Mayhew observed that working-class men were ‘jealous in the extreme’ and ‘To see a man talking to their girl is sufficient to ensure the poor thing a beating.’^37^ Some social reformers likened the relationship between husbands and wives to that of ‘master and slave’;^38^ an analogy associated to ‘The notion that a man’s wife is his PROPERTY in the sense in which a horse is his property … [e]very brutal-minded man, and many a man who in other relations of life is not brutal, entertains more or less vaguely the notion that his wife is his *thing*.’^39^ That wives were possessions was linked to jealousy in literature, but it was not the working-class man who was depicted here. In Eliza Lynn Linton’s novel *Sowing in the Wind* (1890) the protagonist St John’s jealousy is borne out of his possessiveness towards his wife, Isola, and in *He Knew He Was Right*, a thoroughly insane Trevelyan told Emily’s father, ‘She is my wife; my wife; my wife’ and hissed ‘she is mine, mine, mine!’^40^

William Reddy has explored how emotions are the expression of large changes in society, and in her study of jealousy in nineteenth-century France, Masha Belenky argues that discussions of jealousy manifested during times of social change.^41^ This was also the case in Britain where the ‘epidemic of jealousy’—so-called by *Reynolds’ Miscellany* in 1863—coincided with changes in attitudes towards women.^42^ During the latter half of the eighteenth century there was growing revulsion towards domestic abuse due to Britain’s ‘civilising offensive’.^43^ By the 1830s, working men condemned domestic violence as ‘part of a larger effort to reform working-class culture’, and from the 1840s higher conviction rates demonstrated increased outrage towards the killing of women.^44^ Indeed, it was a time when men were increasingly expected to control their passions.^45^ Fictional portrayals of jealousy in marriage were conspicuously associated with debates on Women’s Rights. Newspaper reports detailing infidelity and divorce in England following the Matrimonial Causes Act (1857) which placed the power of the husband, as well as wives’ adultery, under public scrutiny in the Divorce Court, exacerbated the fear of losing possession of one’s wife.^46^ In *He Knew He Was Right* Trevelyan was ‘mad on the subject of his wife’s infidelity’:

His mind was at work upon it always. Could it be that she was as base as this—so vile a thing …? But there were such cases. Nay, were they not almost numberless? He found himself reading the papers for records of such things from day to day.^47^

Greater freedom for wives and increasing matrimonial rights appeared in fiction to provoke husbands into jealousy.

Journalists and authors of fiction addressed changing social codes surrounding marriage and jealousy, and it was through the press and popular literature that they taught readers how to conceptualise violence.^48^ Literature promoted the message that men no longer had the right to be possessive and jealous, and women should no longer regard their husbands as their superiors. Trevelyan struggled with these changes. He wanted ‘[t]o be Master in my own house, and to be paramount in my influence over her’; he wanted ‘submission to my will, which is surely a wife’s duty’.^49^ Trevelyan remains committed to the traditional view of marriage that featured the husband as a source of control in the family.^50^ On the other hand, in the short story ‘How He Was Cured’, the protagonist Herbert Brookes realises the absurdity of his jealousy in a dream which ‘gave him a new idea on the subject of women’s privileges, and on the absurd mistakes jealous husbands are apt to make’.^51^

Authors of fiction used two techniques to communicate their message. The first was the use of *Othello* to depict the detrimental effect of jealousy.^52^ The second was what Belenky terms ‘jealous voyeurism’ (the ‘secretive looking by a jealous lover’).^53^ Belenky found that in nineteenth-century French fiction the jealous voyeur aimed to end the anxiety caused by their uncertainty about their beloved’s faithfulness.^54^ The same can be said of British fiction. In ‘Caleb’s Jealousy’, spying resolved Caleb’s jealousy because he realised his suspicions were foolish. On the other hand, jealous husbands tended to end up insane or dead, their jealousy unresolved.^55^ This distinguished British literature from French where authors tended to end with the brutal murder of the beloved at the hands of their spouse.^56^

## Jealousy in Medical and Scientific Literature

An examination of Victorian medical texts and articles published in *The Lancet* and the *Journal of Mental Science* demonstrates that in comparison to authors of fiction and journalists, alienists and moral philosophers had little to say about jealousy until the late nineteenth century, and even then some physicians perceived a lack of discussion of the emotion. In 1898 physician William O’Neill wrote: ‘Among the … multifarious cases of disease reported year after year in the columns of THE LANCET I have not seen a case of that very old and commonplace complaint, jealousy’.^57^ Nineteenth-century alienists certainly appear to have shied away from discussing the passion in detail. There were exceptions, of course. In 1847, Hanwell asylum physician John Millingen wrote about jealousy, and in the 1850s some medical men wrote about jealousy in journal articles, usually when discussing criminal trials.^58^ It was not until the 1880s and 1890s that alienists, including George Henry Savage and Henry Maudsley, wrote directly about the passion, and not until 1892 was a description of jealousy included in Daniel Hack Tuke’s *A Dictionary of Psychological Medicine*.^59^

General discussions of passions and emotions are more commonly encountered than explicit discussions of jealousy. Maudsley highlighted disordered emotion as a distinct psychological problem, and some physicians warned that it was necessary to restrain the passions and emotions to avoid insanity.^60^ George Robinson wrote that strong passions and emotions ‘depress and harass the feelings, disorder the imagination, and disturb the judgement, until the mind often gives way beneath the pressure of its own woes’.^61^ In such descriptions jealousy is a firm cause of insanity. Alienists and physicians, like journalists and social commentators, believed men must control their passions. Other alienists, including Savage, believed that jealousy ‘may occur alone as a symptom of insanity, or combined with other evidences of mental disorder’.^62^ In this context jealousy was linked to disorders including delusional insanity and monomania.^63^

Some alienists discussed the class and sex of the jealous type. Millingen believed jealousy affected all classes but that working-class men were particularly susceptible:

The fact is that in polite life and in refined society, every one performs a part in a masquerade—seeking to conceal deformities, and mystify his fellow-maskers. The poor cannot afford a mask; … they are only disguised in liquor: uneducated, abandoned, they truly may be called ‘natural children’; their only check is fear—their only guide, their reckless passions.^64^

Echoing the commentaries of social reformers who observed the working-class background of jealous and abusive men, Millingen believed that working-class husbands lacked the self-control to stop themselves committing ‘ferocious deeds’ against their wives when they suspected infidelity, whereas ‘it is beneath the dignity of a well-bred man to ill-use a woman by violent acts’.^65^ Millingen’s association of class and jealousy was not a common feature of medical discussions.

By comparison, the gendering of jealousy was discussed in detail. In February 1898, Harry Campbell, editor of the *Practitioner*, wrote that although jealousy ‘is equally developed in either sex’ before marriage

after marriage it is much more pronounced among the women … not because they have a greater disposition to it but provocation is greater in their case. … [M]an is descended from polygamous ancestors and to pretend that he does not still retain a large measure of the polygamous instinct is to wilfully shut one’s eyes to facts.^66^

As in France, jealousy was described similarly to hysteria.^67^ Savage believed the passion manifested in women more so than men, and associated it with marriage, pregnancy and the menopause, as did Maudsley.^68^ Some alienists and men of science acknowledged male jealousy—Savage in a discussion of young men and Charles Darwin in his discussion of the jealous type—but jealousy was primarily associated with women.^69^

Insane jealousy, like all other forms of insanity, was presumed to have either a ‘moral’ or ‘physical’ cause.^70^ Alienists commonly associated jealousy with hereditary insanity, congenital defects and with physical defects of the body. Savage cited masturbation, impotence and diabetes as causes.^71^ As shown, authors of fiction normally cast jealousy as a disease caused by the imagined indiscretion of a lover or spouse, and Savage similarly blamed jealousy on the ‘artificial relationships of society’ such as marriage, where the absence of a spouse ‘may lead to ideas of neglect, which grow into insane jealousy’.^72^ Thomas Clouston and Maudsley also linked jealousy to suspicion.^73^ Such observations belonged to wider discussions on morbid introspection; the ‘turning of the mind inwards upon itself to the exclusion of external impressions’.^74^ Alienists including Clouston, Maudsley, Charles Mercier, Daniel Hack Tuke and William Bevan Lewis, all warned that ‘the continued Attention upon a certain idea gives it a dominant power’ over the mind and the body.^75^ It was assumed that affected men lost their self control, leading to acts they knew were wrong, including murder.

Like journalists and authors of fiction, some alienists associated sexual jealousy with possession and the fear of losing a partner to another.^76^ Millingen believed that jealousy was the desire to

possess *solely* what we value and enjoy, accompanied with a constant source of uneasiness from the fear of being deprived of that possession, and which engenders a feeling of … hostility towards any person whom we may think likely (probably or improbably) to despoil us of it.^77^

In his *Descent of Man* (1871) Darwin suggested that sexual jealousy was common amongst males of all species. Contrary to Harry Campbell’s argument that men were innately polygamous, he observed that sexual jealousy prompted each male to want to possess his own female partner, and so argued that monogamy was innate to man and other animals.^78^ In the second edition of the text Darwin described the wars that had been occurring for generations between men motivated by their desire to possess women.^79^ Violent sexual aggressiveness was a biologically male-linked trait; it was, as Wiener writes, ‘“a man’s crime” *par excellence*’.^80^ This puzzled some physicians who echoed the assertions of social commentators, authors of fiction and journalists that feelings of possession had no place in marriage. To Campbell it indicated that man had not fully evolved.^81^

Alienists associated jealousy and murder more directly than authors of fiction. To them, jealous individuals witnessed infidelity in the most innocent circumstances. Maudsley wrote that a suspicious husband ‘finds arguments of confirmation in the most innocent circumstances, which he torments himself … with persistently spying into and misconstruing … [he] is blind to the plainest proofs of his unreason’.^82^ Thus contrary to the depictions of authors of fiction, acts of jealous voyeurism pulled men further into the depths of madness, leading to assault, homicide and suicide, rather than resolution. Other alienists also drew upon the darkest themes related to jealousy, including madness and murder, and quoted the Bible and *Othello*, to warn against its potentially devastating results. William O’Neill quoted the Song of Solomon: ‘Jealousy is cruel as the grave; the coals thereof are coals of fire which hath a vehement flame.’^83^ And Savage wrote:

Some young men … make foolish offers of marriage and will not be discouraged, they may murder the girl in a fit of insane jealousy. … In married men insane jealousy is frequently associated with feelings of loss or desire … [and] once start the idea as in the mind of Othello, and no one can tell how, under favouring conditions of mental instability, the unreasonable growth may develop.^84^

The work of British alienists tended to echo French views, such as those expressed by Auguste Debay who defined jealousy as ‘the most ferocious of passions; it drives mortals to all possible excesses, to madness, to suicide, to murder!’^85^ Some contemporaries would have been horrified at such similarities. Conley and Wiener both suggest that the association of crimes of passion with Europeans explains the high conviction rate of jealous husbands who murdered their wives.^86^ Wiener writes that intolerance towards crimes of passion was a ‘marker of British identity and superiority’ and late-Victorian newspapers complained that French juries habitually acquitted both husbands and wives who murdered their spouses.^87^ But jealousy was a crucial factor in some English cases. In 1863, a writer for the *Saturday Review* remarked ‘it seems never to occur to any one that jealousy is the English social vice par excellence’, and argued that contrary to popular opinion, jealousy ‘is developed in a thousand minute forms throughout English society to an extent hardly dreamt of abroad’.^88^ In his *The Criminal* (1895) Henry Havelock Ellis acknowledged the existence of ‘criminals of passion’ in Britain, although the phrase was rarely used elsewhere.^89^ Crimes of passion thus existed (to some) in theory. Yet in late-Victorian Britain insane jealousy and provocation (particularly infidelity) were rarely accepted as a defence.

## Real-life Crimes and Trials

Newspapers regularly reported supposedly doting men murdering (or attempting to murder) their wives and sweethearts, and street literature often explicitly displayed male jealousy.^90^ In the cases of men committed into Broadmoor, Anthony Owston declared his love for his wife before and after murdering her, and Frederick Marshall was reportedly deeply in love with Laura before he stabbed her through the heart.^91^

The question of motivation arises. Trollope’s *He Knew He Was Right* portrayed the visibility of female infidelity in the press as exciting men’s jealousy. Whether this influenced Broadmoor’s patients is questionable. In none of the cases examined did patients mention press reports when explaining their crime and their medical certificates suggest that their literacy levels were low in comparison to Broadmoor’s generally literate population.^92^ They may, of course, have heard others discussing stories of divorce and infidelity. Anthony Owston’s case fits into wider contemporary ideas of jealousy and possession discussed in literature and medical treatises. Owston was committed to Broadmoor after he murdered his wife, Jane. The press reported that Owston was at home with Jane and her mother when, ‘distressed’ and tearful, he told his mother-in-law: ‘I cannot live without Jane, I loved her as a boy and I love her as a man.’^93^ Owston followed Jane upstairs and murdered her. Moral philosopher Alexander Bain described the pain of losing loved ones as ‘deep and intense’ and detailed the ‘misery and distress’ that followed.^94^ Owston seemingly epitomized this. He was represented in the press as wretched; he refused to speak at the inquest but reportedly wrote on a piece of paper: ‘I loved her dearly … She said she would leave me … I’ve been jealous some time … I have been certain they intended to run away. I am guilty, and I hope she is dead … Let me die. I’ve begged of her to live with me, as I could not give her up.’^95^ Wiener shows that as the nineteenth century progressed, instances of wife murder after wives had threatened to leave or had left their husbands increased. The spread of companionate domesticity, which made husbands dependent on the love and support of their wives, contributed to this.^96^ Representations of Owston in the press support this conclusion; he appeared fraught at the apparent failure of his marriage and home-life.

The fear of losing their wives to another man played on the minds of others committed to Broadmoor. In 1881, 73-year-old James Hughes attempted to murder his ‘much younger’ wife whilst they were both inmates of a workhouse in Holloway. A reportedly jealous Hughes had been violent towards her on multiple occasions because he believed she had been having an affair with one of the officials.^97^ In 1884 George Longmore voluntarily readmitted himself into Broadmoor. Longmore’s brother offered the asylum’s Superintendent David Nicolson [Superintendent 1886–1896] an explanation:

He married a very young girl for his wife, apparently very unsuitable for a wife, she had had a farmer’s acquaintance, but had given him up for George. Since their marriage he has occasionally found his wife and her former acquaintance standing near his home laughing, and jesting, after which he appeared very much depressed. To this I attribute as being the chief cause for his returning to Broadmoor.^98^

Even if couples were not married or living together some men seemingly believed women were their possessions.^99^ At the end of the eighteenth century young people began to court who they liked rather than who their parents thought best.^100^ Relationships based on romantic love seemingly presented new opportunities for jealousy. Before Frederick Marshall murdered Laura Wilson he had written her and her father many letters. They were read out at the inquest and printed in the press where they were represented as indicative of Marshall’s insane jealousy.

Marshall’s letters suggest that he wanted sole possession of Laura: he signed them, ‘From your ever true lover’ and ‘I remain your only lover’.^101^ One late-Victorian advice manual depicted the desire to ‘own’ one’s sweetheart as being full of pleasure:

there is that … delight of the courting days—the delight which comes from the enchanting anticipation of possession. … Each sweetheart is consumed with a devouring ambition to own the other. … ‘My beautiful, *my own*,’ exclaims the poetic Lothario, and every lover wants to follow him with the exclamation.^102^

But Marshall’s letters did not contain the jokes and joy typical of love letters.^103^ Rather, Marshall’s declarations of love were juxtaposed with threats, blackmail and angst. It was established at the inquest and subsequently reported in the press that Marshall and Laura had a sexual relationship. This intimacy seemingly aggravated Marshall’s feelings of ownership and he wrote Laura the following letter:

[Y]ou are a deceitful to [sic] faced young woman. You said in bed you’d be true to me. … If you won’t see me on Sunday I will see Charlie and tell him I have been with you and slept with you 4 nights, and your father I will write … and tell him the same … if you mean to not have me tell me and have no one else and if you let me sleep [with you] … one more night I will say nothing … if I don’t see you or hear from you before Wednesday night I will do as I say. I feel nearly mad to think all our days and hours are spent all for nothing. I made up my mind to have you … you ought to be afraid of dropping dead the oaths you’ve taken always to me. … Write soon dear Laura (a number of crosses)

From your ever true lover

Fred (three crosses)^104^

The inquest heard that Laura’s father thought Marshall was ‘peculiar’ and warned him to stay away from their home, but he took little notice and began scaling their garden wall to engage in acts of jealous voyeurism: ‘You got up [to] his concertina to play … and he went out to get some beer and when he came in you was laughing and talking with him. … I was over the fence watching you, … [if] I see you in there stopping while his [sic] in there by the Heaven above I’ll swing for you.’^105^ Unlike his fictional counterparts, Dougauld and Caleb, Marshall’s spying reportedly exacerbated his jealousy and made him increasingly threatening, as Maudsley believed it would. Marshall told Laura’s father, ‘I mean to have her … I know no one else will’, and began to end his letters with remarks such as ‘Change your love once more to me dearest or I am lost to the world … You ought to be burnt if you’ll not change.’^106^ Marshall associated these violent thoughts with his mental state and told Laura: ‘I am not very well I feel nearly mad when I think of you.’^107^

Following the inquest, Marshall was bound for trial at the Old Bailey and transferred to Clerkenwell Gaol. The Prisons Act (1865 and 1877) stipulated that all prisoners in England and Wales had to be regularly subjected to a medical inspection. Clerkenwell’s Prison Medical Officer (PMO) found Marshall ‘does not understand the gravity of the crime’, and has ‘no comprehension of moral obligation’.^108^ The PMO forwarded his report to the Home Secretary, William Harcourt, who ordered two physicians to examine Marshall. They found him insane and he was sent to Broadmoor.^109^

The press solidified the link between jealousy, madness and murder that was implied by authors of fiction and written about by some alienists and by Marshall. In the case of James Hughes, the *Standard* reported that his ‘conduct had been generally quiet, except when he gave way to paroxysms of rage on account of the delusion he entertained with regard to his wife’s conduct’.^110^ A journalist assumed that William Lloyd ‘must have gone suddenly mad’ through jealousy before he murdered his wife.^111^ As in fiction, wife-murderers were represented in the press as wild, strong and irrational, their crimes couched in the language of insanity and brutality. Philip Dawe reportedly murdered his wife ‘by beating her about the head with a Russian club’ ‘with immense force’, and Matthew Cook ‘threw [his child] over the hedge into the gutter’ before he ‘barbarously murdered his wife by cutting her throat from ear to ear’.^112^

It has been shown that jealousy was entrenched in Victorian culture, but press reports and trial proceedings suggest that when it came to medico-legal discourse the passion was marginalised. Two types of defence were employed in jealous murder cases: provocation and insanity. Some contemporaries associated provocation and homicide.^113^ It was commented in the *Journal of Mental Science*:

To the ordinary average man the motive of jealousy is not enough to prompt the comparatively trivial act of murdering the woman of whom he is jealous. Yet in some men this motive does prompt to this action, and by universal consent the action is considered as sane and as sufficiently accounted for by the motive.^114^

To some, provocation was an acceptable defence in certain circumstances: men had to lose their self-control at the right time and for the right reason. In 1866, eminent English judge, James Fitzjames Stephen, drew upon the early-modern conception that for an act of provocation to be acceptable it had to be committed suddenly when there is ‘no time for the blood to cool between the offence and retaliation’.^115^ Roger Chadwick writes that tales of provocation sometimes ‘touched the hearts of the male juries, judges and civil servants involved’.^116^ Consequently, men were sometimes convicted of manslaughter rather than murder, and thus spared the gallows.^117^ Some laymen shared the view that provocation justified a man’s loss of self-control, suggesting that reality did not always reflect the ideals presented in literature, the press and the works of social commentators. In November 1888 a friend of Broadmoor patient and wife-murderer Timothy Grundy wrote to David Nicolson:

he was more to be pitied than blamed, it was his foolish wife that provoked and tantalized him beyond endurance, would not [keep] company with him and would flirt and joke with other men. He [was] a hearty man just in [the] prime of youth, just when the passions are strongest … to be treated so unreasonably and outrageously wrong it was maddening beyond human endurance it was not insanity it was being sane enough to feel his wrongs to keenly.^118^

Nevertheless, scholarly research suggests that as the nineteenth century progressed, judges and juries hardened towards pleas of provocation in jealous murder trials.^119^

Defence lawyers increasingly focused on defendants’ state of mind to avoid execution. An examination of legal and medical literature, however, suggests that cases involving jealousy were not straightforward. Distinguishing between normal and morbid jealousy was seemingly problematic, and discussion arose about how strong jealousy had to be before a defendant could be declared insane.^120^ The extent to which passion indicated insanity was also questioned. In 1896 it was declared in the *Journal of Mental Science* that passion should never be used as a defence:

It is a matter of common knowledge that when rein is given to passion, whether bloodthirstiness or lust … the appetite grows with its indulgence, and, in his gathering frenzy, the agent plunges into excesses which he had at first never contemplated. … Mad he may be in one sense—in the sense in which the maxim *ira furor brevis est* is to be understood—but if such an access of frenzy is to be held an excuse for crime, we may as well abolish our criminal jurisprudence altogether.^121^

The following year, Maudsley wrote that passion could be used as evidence of insanity in court but highlighted:

When an insane person is on trial … it is commonly taken for granted by lawyers that if an ordinary motive for the act, such as anger, revenge, jealousy or any other passion can be discovered there is no ground for alleged insanity or … exemption from responsibility by reason of insanity.^122^

This certainly appears to have been the case.

French juries openly accepted insane jealousy and provocation as legitimate causes of murder, and in the late nineteenth century several American trials involved men who had killed either their wives or their wives’ lovers who, it was successfully argued, ‘suffered from a legitimate jealousy that simply overcame their will’.^123^ Daniel McFarland’s defence argued that jealousy had induced temporary insanity and told the court:

jealousy is the rage of a man. … Those who dishonor husbands are here warned of their doom. … Jealousy, which defies and bears down all restraint, whether it be what we technically call insanity or not, is akin to it. It enslaves the injured husband, and vents itself in one result, which seems to be inevitable and unavoidable.^124^

Such direct references to jealousy in English courtrooms rarely occurred. Although jealousy was referred to and implied, it was not always used as proof of insanity.

An examination of newspaper reports and trial proceedings suggests that during an Englishman’s trial, jealousy was either ignored or referred to by the press or by lay witnesses who made no explicit connection between the passion and insanity. This analysis also highlights the use of some interesting defence tactics, particularly the desire to find evidence of insanity in places other than the crime, as Wiener and Roger Smith found in their respective studies of violent men and Victorian trials.^125^ Following the murder of his wife, Owston attempted suicide and was taken to hospital. The inquest heard that whilst hospitalised, Owston’s ‘mental condition became so much worse—he being subject to fits of despondency’. His ‘strange conduct’ reportedly ‘terrified’ the other patients and he was transferred to the lunatic wards at the Bradford Workhouse.^126^ Owston’s defence argued that he was unfit to plead and that the evidence was sufficient to show his mind was unsound. The jury agreed.^127^ Owston’s self-confessed jealousy was thus ignored and evidence of his insanity found elsewhere. This was not unusual.

In 1866, Daniel Beloe murdered the woman he was living with.^128^ A witness to the crime told the inquest that ‘he was sure jealousy was the cause of the act’ and the press reported that Beloe said it had ‘all been done through love and jealousy’.^129^ At the trial a witness stated that when he was discovered at the scene, Beloe ‘put his hand to his heart’ and ‘said that he did it through jealousy’.^130^ Following the murder, Beloe attempted to commit suicide and was taken to Guy’s Hospital where Savage examined him. When questioned in court, Savage made no reference to jealousy, although he would consider jealousy a potential cause of insanity 30 years later. Instead, he told the courtroom that a ‘violent injury to his brain’ might have affected Beloe’s ‘mental powers’.^131^ Beloe’s defence subsequently called witnesses to show that he had previously suffered a head injury that had led to excitement, intemperance, idleness and suicidal thoughts.^132^ He was found not guilty on the grounds of insanity.^133^

Establishing whether a defendant was delusional on the subject of his wife’s infidelity was one way alienists, judges and juries sometimes determined the insanity of a jealous individual. To satisfy the McNaughton Rules (1843) a defendant must be shown to be suffering from delusions. In 1855 John Charles Bucknill wrote:

If the jealousy had any foundation in real occurrences, it might be the natural feeling of an outraged husband, in which case its fatal result would be wilful murder, punishable by death. On the other hand, this feeling of jealousy might be a symptom of insanity, and the circumstances upon which it was founded might merely be the creation of a diseased brain, in which the fatal act would be the result of mental disease, and as such ought not to be punished.^134^

In Owston’s case it was established that his wife had been having an affair and had planned to leave him. Thus, although Owston’s insanity had been demonstrated by descriptions of his strange behaviour he was not legally mad. An examination of such trials lends support to the argument put forth by historians including Eigen and Smith that the McNaughton Rules were followed haphazardly and each trial was judged differently.^135^ In theory, the Rules had set a high standard for insanity, the inability to understand the nature of the act committed, yet in some cases in which jealous wife murderers were committed insane to Broadmoor they were seemingly aware of what they had done; as discussed, Owston wanted to be hanged and was ‘glad’ Jane was dead.

Conley shows that between 1867 and 1892 only five Englishmen accused of killing women who rejected them were found insane; 33 were executed.^136^ That jealousy had a history of failure in the courtroom may explain why defence lawyers bypassed jealousy and instead sought evidence of insanity elsewhere.^137^ In addition, lawyers may not have invoked jealousy because the notion that the crime was the result of passion was open to dispute. Thus they either failed to acknowledge jealousy existed or linked it to a recognisable form of mental disease that could be seen as stopping the defendant from forming criminal intent. The medicalisation of intemperance in the 1870s helped in this regard and the insanity of some jealous murderers committed to Broadmoor was attributed to *delirium tremens* (DT). The press reported that ‘a fiendish passion’ had taken hold of Matthew Cook before he murdered his wife and he was represented as an obsessive husband who had engaged in acts of jealous voyeurism.^138^ At Cook’s trial, the defence used DT and evidence of a hereditary taint to convince the jury he was insane.^139^ Although some alienists associated alcoholism with jealousy, and journalists explicitly referred to Cook’s ‘jealous’ disposition, no reference to the passion was made during his trial.

Jealousy was increasingly condemned in popular literature and newspaper articles, but its complexity also likely deterred lawyers from relying upon the passion as a mitigating factor in murder trials. As shown, defining jealousy—its causes and symptoms—was not straightforward, and medical explanations of the passion were sometimes at odds. Jealousy was represented as both a cause and a symptom of insanity, and even in those cases where jealous insanity was suspected it was, as alienist George Fielding Blandford declared, ‘difficult to certify’.^140^ In both popular and medical literature it was represented as a dangerous passion leading to many different emotions, including anger, rage, fear and sadness, all of which were present in the real-life cases examined in this article. Jealousy is (and was) considered a feeling consisting of many emotions with no distinct expression.^141^ It was feared that such a strong passion could, if left to fester, result in the loss of control and murder. Some social commentators and authors of fiction associated this loss of control with the working class, and whilst physicians and alienists rarely explicitly mentioned class in their discussion of the passion, when it was featured it was linked to biological weakness and the inherent inability of working-class men to control their ‘reckless passions’.^142^ In the courtroom, despite the testimony of laymen and the assertions of some defendants that jealousy played a role in their crime, medical witnesses appear to have overlooked the passion as a cause of insanity, instead choosing to rely on causes such as bangs to the head and poor heredity. The same thing happened at Broadmoor.

## Broadmoor

It has been shown that jealousy was openly discussed and condemned in the British press and popular literature, and during the latter decades of the nineteenth century its visibility in the medical literature increased. Yet references to the emotion in the Broadmoor case files are rare. Before a patient was transferred from prison to Broadmoor, the PMO completed ‘Schedule A’; a medico-legal document which included information about the patient’s crime, trial, verdict and the cause of insanity. The cause of insanity PMOs recorded tended to correspond to the observations made by the medical witness at the trial, rather than a patient’s own explanation of the criminal act, or that of his friends and family; the jealous frenzies depicted in numerous newspaper reports are also absent. In the case of Beloe, the cause of insanity was the ‘blow on the head’ as Savage described, rather than Beloe’s self-confessed jealousy, which lay witnesses had described. In both Matthew Cook’s and William Lloyd’s cases the consumption of alcohol was the perceived cause of insanity, rather than the jealous frenzy the press described. Of course, given that jealousy was not a medico-legal category it is not surprising that it rarely appeared on Schedule A. Jealousy was recorded as a cause of insanity on Schedule A in the cases of only two of the 45 men committed to Broadmoor for the attempted murder or murder of their wives or sweethearts, both in 1885: Joseph Cantrill and John Willoughby.^143^

Jealousy remains strikingly absent when patients’ medical files are examined in more depth. Even in the cases of the defendants discussed earlier, whose alleged jealousy was explicitly addressed in the press or in some instances at their trials (sometimes by defendants’ themselves), Broadmoor’s medical officers made no association between their alleged jealousy and insanity. In 1890 the cause of Anthony Owston’s insanity was recorded as ‘domestic troubles’.^144^ Yet, his case file contained a cutting of a newspaper article from 1889 that reported that ‘in a frenzy of exasperation’ Owston had murdered his wife, and in his ‘jealousy’ had attempted to murder his rival.^145^ In Hughes’ case, the Governor of Newgate Prison wrote to Superintendent William Orange (Superintendent 1870–1886) informing him that Hughes was ‘jealous of her [his wife]. He was under the delusion that she had been unfaithful to him.’^146^ Jealousy is absent from the medical reports in James Hughes’ case file. According to medical reports, William Lloyd was epileptic and maniacal, and Matthew Cook was intemperate and had a predisposition to insanity: in both of their cases, journalists deemed jealousy to be the dominating factor. In John Willoughby’s case, the cause of his insanity was ‘hereditary predisposition’ rather than the ‘jealousy’ recorded on Schedule A.^147^

Frederick Marshall’s case is the most striking example in which the jealousy that was so overtly discussed in the press and at the inquest was seemingly overlooked. Superintendent Nicolson was apparently aware of the circumstances surrounding the case; indeed, he had filed 12 pages of newspaper cuttings relating to it.^148^ To Nicolson, Marshall’s behaviour was explained by imbecility. He believed Marshall was ‘weak-minded from birth’ and had a hereditary predisposition to insanity.^149^ He was also irritable, easily offended, petty and childish.^150^ Nicolson’s evaluation of Marshall was typical of late nineteenth-century psychiatry which, influenced by the evolutionary models of Herbert Spencer, increasingly focused on neurological rather than psychological models of insanity.^151^ Victorian neurologist, John Hughlings Jackson, adopted a three-tier hierarchy of the nervous system.^152^ According to this model, insanity led to the loss of the highest nervous centres, resulting in a loss of control and inhibition. Echoing the Jacksonian model, Nicolson depicted Marshall as a man whose nervous system had not fully developed; he was missing the highest level and was thus incapable of exercising control: with ‘approaching manhood the balance of his character and of his appetites and passions has … been greatly disturbed and not got beyond the control of his volition. His capacity for reflection and his power of will for self guidance have not been kept in pace with his added years.’ Nicolson appealed to both moral (untamed passions) and physical causes of insanity:

He suffers from a moral inability and incapacity which, under ordinary circumstances, need not cause him to act of intemperately or insanely; but which, under any feat of strain or excitement and appeared by the severe headache to which is so liable, end in … active and dangerous insanity and irresponsibility.^153^

Nicolson’s report contains similar observations to those made in his article ‘The Morbid Psychology of Criminals’ (1875) in which he reported that the will of weak-minded (imbecile) men ‘is without energy; it is incapable of exertion; it cannot act; it is paralysed’.^154^ What we see at Broadmoor is discussion of the physical causes and symptoms of insanity alienists described in their published works on the passions: melancholia, delusional insanity, mania, intemperance, hereditary predisposition to insanity, and imbecility all appear in the cases of men who were, according to the press, lay testimony or themselves, jealous.^155^

This is not entirely surprising. By the end of the century alienists tended to attribute insanity to either predisposing or exciting causes. The former were usually biological and linked to a poor heredity, whereas the latter were the result of some moral trigger such as ‘domestic trouble’. An insane murderer could have been seen to have a biological weakness that made him more susceptible to being overwhelmed by his passions and emotions, as Nicolson reported Marshall had been. However, it is noteworthy that Marshall’s case was the only instance where Broadmoor’s medical staff directly associated physical disorders with the passions. In other cases jealousy was seemingly disregarded as the cause of criminal insanity. Philip Dawe was committed to Broadmoor in 1881. In his Annual Report for that year, William Orange wrote, ‘[Dawe] says he was not married to the woman; that they had lived unhappily for some time; that she was not well-conducted, and had frequently given him cause to be jealous.’ Orange instead attributed Dawe’s insanity to his drinking, sleeplessness and low spirit, and noted that he ‘had been in an asylum before; had several times attempted suicide, and had relations in asylums.’^156^

At Broadmoor, as in some medical treatises, jealousy was seemingly considered a symptom rather than a cause of insanity. Two reasons might be suggested for this. First, medical staff may have adhered to the broadly ascribed (medical) notion that jealousy was primarily a female condition. Perhaps, then, we can see glimpses of attempts to separate female causes of insanity (strong passions and emotions) and male causes of insanity (primarily physical) within an asylum setting. The second reason why Broadmoor’s staff cast jealousy as a symptom rather than a cause of insanity may have been that the notion that insanity had a physical cause was, as Andrew Scull has noted, a vital prop’ for alienists’ contentions that insanity was fundamentally a medical problem.^157^ It might be that providing a physical (and scientific) explanation for the cause of criminal insanity enabled asylum doctors to distinguish medical theories from popular ideas. This supports Akihito Suzuki’s argument that there ‘existed a contest for interpretive authority over the act of decoding and defining disease, or what Katherine Hunter has called “a silent tug-of-war over the possession of the story of illness”’.^158^ This is not only evident between medical and popular representations of disease; Dawe’s case suggests the conflict and hierarchical relationship that existed between doctor and patient, the latter having no control over their diagnosis at Broadmoor.

## Conclusion

Male jealousy was often discussed in the popular press and in fiction, and by laymen and, to a lesser extent, within the courtroom and medical literature in Victorian Britain. To some extent jealousy was amorphous, with contradictory representations presented in these different arenas, but some commonalities existed. Two distinct causes of male jealousy have been highlighted: jealousy caused by emotions (hurt feelings) and jealousy caused by a perceived violation of property. While clearly demarcated, they were equally dangerous. Some authors of fiction, social commentators, and journalists shared these understandings and represented jealousy as a negative passion that was liable to cause madness and violence. At least until the end of the nineteenth century, jealousy was less prevalent in medical literature, but alienists’ discussions regarding the causes and results of jealousy were sometimes mirrored by those found in popular literature and the press. However, some alienists considered jealousy to be a symptom of disorder rather than a cause.

An examination of the sources indicates that ideas about jealousy crossed national borders in the nineteenth century.^159^ Both British and French authors of fiction depicted the brutal and insane nature of the jealous man, even if their stories tended to end differently. Ideas about jealousy crossed not only spatial boundaries but temporal boundaries too, and this research supports Gesa Stedman’s findings: both authors and alienists frequently referred to Shakespeare as a source of authority.^160^ Where there is no similarity, in space or time, is in the courtroom. This is because different national social and legal contexts make for different emotional regimes. The association of jealousy and crimes of passion with ‘uncivilised’ Europeans demonstrates this, as do changing ideas regarding the relationship between men and women in Britain, as demonstrated through an examination of popular literature and press reports. In Britain, unlike France or America, there was not a ‘crime of passion’ defence, meaning an appeal to jealousy was of no use in criminal proceedings. The medico-legal emphasis on physical and hereditary explanations of disease in insanity defences meant that these, rather than feelings or emotions, were given centre stage in the courtroom.

Popular ideas and some medical ideas about jealousy did not permeate Broadmoor’s walls. The asylum’s staff appears to have bypassed the passion, even when PMOs, the press or patients themselves referred to it explicitly. Three reasons might be suggested for this. First, asylum doctors may have ignored male jealousy because they believed the passion primarily affected females. Secondly, it might be that whilst jealousy was not explicitly discussed, the passions and emotions were presumed to excite an underlying biological predisposition to insanity, although explicit evidence for this (other than in Frederick Marshall’s case) is lacking. Thirdly, while jealousy was sometimes the result of misbehaviour by a patient’s sweetheart, by focusing upon physical diseases the asylum’s staff placed these patients within their sphere of authority. In popular literature, jealousy was remedied through the realisation that a suspicion was wrong, but in real-life cases jealousy seemingly required an underlying physical cause to justify treating the patient in Broadmoor.

## Footnotes

1 ‘Murder by a Disappointed Lover: A Girl Stabbed to the Heart’, *Western Mail*, 23 December 1884.

2 ‘The Woolwich Murder’, *The Morning Post*, 6 January 1885, 2.

3 ‘The Woolwich Murder’, *The Standard*, 24 December 1884, 3.

4 Harvey Gordon, *Broadmoor* (London: Psychology News Press, 2012); Deborah Weiner, ‘“This Coy and Secluded Dwelling”: Broadmoor Asylum for the Criminally Insane’, in Leslie Topp, James E. Moran and Jonathan Andrews, eds, *Madness, Architecture and the Built Environment* (New York and London: Routledge, 2007), 131–48.

5 Louise Hide, *Gender and Class in English Asylums, 1890–1914* (Basingstoke: Palgrave Macmillan, 2014); Pamela Michael, ‘Class, Gender and Insanity in Nineteenth-Century Wales’, in Jonathan Andrews and Anne Digby, eds, *Sex and Seclusion, Class and Custody: Perspectives on Gender and Class in the History of British and Irish Psychiatry* (New York: Rodopi Press, 2004), 95–122; Anna Shepherd, *Institutionalizing the Insane in Nineteenth-Century England* (Oxon: Pickering and Chatto, 2014); Jade Shepherd, ‘“One of the best fathers until he went out of his mind”: Paternal Child-Murder, 1864–1900’, *Journal of Victorian Culture*, 2013, 18, 17–35.

6 For jealousy in American and French history, Peter Stearns, *Jealousy: The Evolution of an Emotion in American History* (New York and London: New York University Press); Masha Belenky, *The Anxiety of Dispossession: Jealousy in Nineteenth-Century French Culture* (Lewisburg: Bucknell University Press, 2008). For recent considerations of the emotions, the History of Emotions Blog: <https://emotionsblog.history.qmul.ac.uk>, accessed September 2013.

7 Carolyn Conley, *Certain Other Countries: Homicide, Gender and National Identity in Late-Nineteenth Century England, Ireland, Scotland and Wales* (Columbus: Ohio State University Press, 2007), 127; Martin Wiener, *Men of Blood: Violence, Manliness, and Criminal Justice in Victorian England* (Cambridge: Cambridge University Press, 2006), 201–15, 230; Stephen Kern, *The Culture of Love: Victorians to Moderns* (Cambridge, MA and London: Harvard University Press, 1992), 264–80; Valerie Pedlar, *The Most Dreadful Visitation: Male Madness in Victorian Fiction* (Liverpool: Liverpool University Press, 2006), 111–33; Ronald Rae Mowat, *Morbid Jealousy and Murder: A Psychiatric Study of Morbidly Jealous Murderers at Broadmoor* (London: Tavistock Publications, 1966).

8 Simon May, *Love: A History* (New Haven & London: Yale University Press, 2011); Barbara Rosenwein, ed., *Anger’s Past: The Social Uses of an Emotion in the Middle Ages* (New York: Cornell University Press, 1998); Joanna Bourke, *Fear: A Cultural History* (London: Virago, 2006).

9 Claire Langhamer, ‘Everyday Love and Emotions in the Twentieth Century’, History of the Emotions Blog, accessed 17 September 2013.

10 Susan J. Matt, ‘Current Emotion Research in History: Or, Doing History from the Inside Out’, *Emotion Review*, 2011, 3, 117–24, 119; Claire Langhamer, *The English in Love: The Intimate Story of an Emotional Revolution* (Oxford: Open University Press, 2013).

11 Between 1864 and 1900, 84% of Broadmoor’s male patients were working class: Jade Shepherd, ‘Victorian Madmen: Broadmoor, Masculinity and the Experiences of the Criminally Insane, 1863–1900’ (unpublished PhD thesis, Queen Mary University of London, 2013), 59–62.

12 Berkshire Record Office (BRO), D/H14/D1/1/1/1, Admissions Register, 1863–1871; D/H14/D1/1/1/2, Admissions Register, 1868–1900. Hereafter, all references beginning D/H14 are from the BRO.

13 Jonathan Andrews, ‘Case Notes, Case Histories, and the Patients Experience of Insanity at Gartnavel Royal Asylum, Glasgow, in the Nineteenth Century’, *Social History of Medicine*, 1988, 11, 255–81, 266.

14 For patients inside Broadmoor, Jonathan Andrews, ‘The Boundaries of Her Majesty’s Pleasure: Discharging Child-Murderers from Broadmoor and Perth Criminal Lunatic Department c. 1860–1920’, in *Infanticide: Historical Perspectives on Child Murder and Concealment, 1550–2000*, ed. by Mark Jackson (Aldershot: Ashgate Publishing, 2002), 216–48; Shepherd, ‘“One of the best fathers until he went out of his mind”’.

15 Joel Peter Eigen*, Unconscious Crime: Mental Absence and Criminal Responsibility in Victorian London* (Baltimore and London: The Johns Hopkins University Press, 2003); Wiener, *Men of Blood*. Work drawing upon OBPO is vast. See, ‘Publications that Cite the Old Bailey Proceedings Online’, <http://www.oldbaileyonline.org/static/Publications.jsp>, accessed 13 November 2015.

16 Joel Peter Eigen, ‘Diagnosing Homicidal Mania: Forensic Psychiatry and the Purposeless Murder’, *Medical History*, 2010, 54, 433–56, 435.

17 Helen Goodman, ‘“Madness and Masculinity”: Male Patients in London Asylums and Victorian Culture’, in Thomas Knowles and Serena Trowbridge, eds, *Insanity and the Lunatic Asylum in the Nineteenth Century* (London: Pickering and Chatto, 2015), 149–63; Roy Porter, ‘“The Hunger of Imagination”: Approaching Samuel Johnson’s Melancholy’, in William F. Bynum, Roy Porter and Michael Shepherd, eds, *The Anatomy of Madness: Essays in the History of Psychiatry*, 3 vols (London: Routledge, 1985–88), VI (1985), 62–88; Joseph Melling, Bill Forsythe and Richard Adair, ‘Families, Communities and the Legal Regulation of Lunacy in Victorian England: Assessments of Crime, Violence and Welfare in Admissions to the Devon Asylum, 1845–1914’, in Peter Bartlett and David Wright, eds, *Outside the Walls of the Asylum: The History of Care in the Community 1750–2000* (London and New Brunswick, NJ: The Athlone Press, 1999), 153–80.

18 David Konstan, *Envy, Spite and Jealousy: The Rivalrous Emotions in Ancient Greece* (Edinburgh: Edinburgh University Press, 2003).

19 Peter Stearns, ‘Jealousy in Western History: From Past Toward Present’, in Sybil. L. Hart and Maria Legerstee, eds, *Handbook of Jealousy: Theory, Research, and Multidisciplinary Approaches* (Chichester: Wiley-Blackwell, 2010), 7–26, 10.

20 Author unknown, *Jealousy Exemplified in the Awful Tragic and Bloody History of the Loves and Untimely Deaths of Othello and Desdemona* (London: Hodgson & Co., 1823); R. N. Hutton, *Jealousy: A Novel* (T. C. Newby: London, 1849).

21 Andrew Smith, *Victorian Demons: Medicine, Masculinity and the Gothic at the Fin-De-Siècle* (Manchester and New York: Manchester University Press, 2004); Jane Wood, *Passion and Pathology in Victorian Fiction* (Oxford: Oxford University Press, 2001).

22 Quoted in Daniel D. Oberhelman, ‘Trollope’s Insanity Defence: Narrative Alienation in *He Knew He Was Right*’, *Studies in English Literature, 1500–1900*, 1995, 35, 789–806, 790.

23 Stearns, *Jealousy*, 28.

24 ‘Jealousy’, *Saturday Review*, 17 March 1894, 275–6, 276.

25 ‘Jealousy’, *Saturday Review*, 7 March 1863, 298–300, 298.

26
*Ibid*., 299.

27 Anthony Trollope, *He Knew He Was Right* (1869) (Oxford: Oxford University Press, 1996), 925.

28 Eliza Peakes, *Jealousy and Revenge Tales* (London: 1845), 21, 285, 294.

29 ‘No, Never!’, *Every Week*, 12 October 1885, 233–5, 234.

30 ‘No, Never!’, *Every Week*, 2 November 1885, 282–3.

31 M. K. D, ‘Caleb’s Jealousy’, *London Reader of Literature, Science, Art and General Information*, 1 April 1876, 525–6, 526.

32 For unrequited love and female insanity, Helen Small, *Love’s Madness: Medicine, the Novel, and Female Insanity, 1800–1865* (Oxford: Clarendon Press, 1996).

33 R. W. Dale, *The Ten Commandments* (London: Hodder and Stoughton, 1871), 63.

34 John William Taylor, *Love, Courtship, and Marriage: How to Read Character by the Walking, Hand-Shaking, etc*. (London: L. N. Fowler, 1891), 41.

35 Conley, *Certain*, 127.

36 Frances Power Cobbe, ‘Wife-Torture in England’, *Contemporary Review*, 1878, 32, 55–87.

37 Henry Mayhew, *London Labour and the London Poor: The Condition of Earnings of Those That Will Work, Cannot Work, and Will Not Work*, 3 vols (London: Charles Griffin and Company, 1861), VI, 46.

38 John Stuart Mill, ‘The Subjection of Women (1869)” in *Essays on Sex Equality*, ed. by Alice Rossi (Chicago: Chicago University Press, 1970), 163–6, 235–6.

39 Cobbe, ‘Wife-Torture’, 62.

40 Eliza Lynn Linton, *Sowing in the Wind* (London: Chatto and Windus, 1890); Trollope, *He Knew*, 736.

41 William Reddy, *The Navigation of Feeling* (Cambridge: Cambridge University Press, 2001); Belenky, *Anxiety.*

42 ‘Jealousy’, *Reynolds Miscellany of Romance, General Literature, Science, and Art*, 7 November 1863, 309.

43 Wiener, *Men of Blood*, 35–7.

44 For working-class perceptions of domestic violence, Anna Clark, ‘Domesticity and the Problem of Working-class Wifebeating in Nineteenth-century Britain: Working-class Culture, Law and Politics’, in Shani D’Cruze, ed., *Everyday Violence in Britain 1850–1950* (London: Pearson Education Ltd, 2000), 27–40.

45 Violence was deemed a natural male trait, and men were expected to control rather than erase their natural aggressive impulses. Judith Rowbotham, ‘“Only when drunk”: The Stereotyping of Violence in England, c. 1850–1900’, in D’Cruze, ed., *Everyday Violence*, 155–69, 159.

46 A. James Hammerton, ‘Victorian Marriage and the Law of Matrimonial Cruelty’, *Victorian Studies*, 1990, 33, 269–92.

47 Trollope, *He Knew*, 361–3.

48 Rowbotham, ‘“Only when drunk”, in D’Cruze, ed., *Everyday Violence*, 157.

49
*Ibid*., 589–90.

50 John Tosh, *A Man’s Place: Masculinity and the Middle-Class Home in Victorian England* (New Haven and London: Yale University Press, 2007), 53–78.

51 W. C. Russell, ‘How He Was Cured’, *Temple Bar*, 1875, 45, 500–15, 514.

52 For the Victorians and *Othello*, Adrian Poole, *Shakespeare and the Victorians* (London: Arden Shakespeare, 2004), 98.

53 Belenky, *Anxiety*, 60.

54
*Ibid*.

55 Kern, *Culture*, 268, 280.

56 Belenky, *Anxiety*, 21.

57 William O’Neill, ‘A Case of Jealousy’, *The Lancet*, 22 January 1898, 223–4, 223.

58 John Millingen, *Mind and Matter Illustrated by Considerations on Hereditary Insanity and the Influence of the Temperament on the Development of the Passions* (London: J. Hurst, 1847); John Charles Bucknill, ‘Trial of Robert Handcock for the Murder of his Wife, Phillipa Handcock, at the Devon Winter Assizes, before Mr. Baron Parke, Dec. 10^th^, 1855. Plea of Insanity’, *Journal of Mental Science*, 2 (1856), 245–53, 245.

59 George Henry Savage, ‘Jealousy’, in Daniel Hack Tuke, ed., *A Dictionary of Psychological Medicine: Giving the Definition, Etymology and Synonyms of the Terms Used in Medical Psychology with the Symptoms, Treatment, and Pathology of Insanity and the Law of Lunacy in Great Britain and Ireland* 2 vols (London: J & A Churchill, 1892), 721–3. Although it was not discussed, ‘Love (love thwarted, jealousy)’ was listed as a cause of insanity in the third edition of Bucknill and Tuke’s, *A Manual of Psychological Medicine, Containing the Lunacy Laws: The Nosology, Aetiology, Statistics, Description, Diagnosis, Pathology, and Treatment of Insanity, with an Appendix of Cases*, 3rd edn (London: J & A Churchill, 1879), 107.

60 Henry Maudsley, *The Pathology of Mind, A Study of its Distempers, Deformities and Disorders* (London: Macmillan and Co., 1895), 349; John Williams, *Insanity, its Causes, Prevention, and Cure; Including Apoplexy, Epilepsy, and Congestion of the Brain*, 2nd edn (London: John Churchill, 1852), 29; William A. Guy, *The Factors of the Unsound Mind with Special Reference to the Plea of Insanity in Criminal Cases and the Amendment of the Law* (London: Thos. De La Rue, 1881), 127.

61 George Robinson, *On the Prevention and Treatment of Mental Disorders* (London: Longman, Brown, Green, Longmans & Roberts, 1859), 136.

62 Savage, ‘Jealousy’, 721.

63 George Henry Savage, *Insanity and Allied Neuroses: Practical and Clinical* (London and New York: Cassell, 1884), 237, 263; John Charles Bucknill, ‘The Diagnosis of Insanity’, *Journal of Mental Science*, 2 (1856), 229–45, 244.

64 Millingen, *Mind*, 384.

65
*Ibid*., 382.

66 Harry Campbell, ‘A Case of Jealousy’, *The Lancet*, 5 February 1898, 402–3, 403.

67 Belenky, *Anxiety*, 49.

68 Savage, ‘Jealousy’, 721; Henry Maudsley, *The Physiology and Pathology of Mind* (London: Macmillan and Co., 1867), 327.

69 Savage, ‘Jealousy’, 722. Darwin showed that ‘All males are jealous, even male salmon, which fight ferociously for the fair, and very funny it is the spectacle, as they drive at each other.’ ‘Jealousy’, *Saturday Review* (1894), 275.

70 ‘Moral’ did not comprise a value judgement on behaviour but rather meant what we would now call ‘psychological’ or ‘psycho-social’.

71 John Milner Fothergill, ‘The Mental Aspects of Ordinary Disease’, *Journal of Mental Science*, 1874, 20, 387–409, 406; Savage, ‘Jealousy’, 722–3.

72 George Henry Savage, ‘The Influence of Surroundings on the Production of Insanity’, *Journal of Mental Science*, 1891, 37, 529–35, 534.

73 Thomas Clouston, *Clinical Lectures of Mental Diseases* (London, J. & A Churchill: 1883), 255; Maudsley, *Pathology*, 327.

74 Michael J. Clark, ‘“Morbid Introspection”, Unsoundness of Mind, and British Psychological Medicine, 1830–1900’, in Bynum, Porter and Shepherd, eds, *The Anatomy of Madness: Essays in the History of Psychiatry*, 3 vols (London: Routledge, 1985–88), VIII (1988), 71–101, 73.

75 Quoted in *Ibid*., 79.

76 Maudsley, *Pathology*, 377; Savage, ‘Jealousy’, 721.

77 Millingen, *Mind*, 377.

78 Gowan Dawson, *Darwin, Literature and Victorian Respectability* (Cambridge: Cambridge University Press, 2007), 75–6.

79 Charles Darwin, *The Descent of Man, and Selection in Relation to Sex*, 2nd edn (London: John Murray, 1879), 627.

80 Wiener, *Men of Blood*, 201.

81 Campbell, ‘A Case of Jealousy’, 403.

82 Maudsley, *Pathology*, 325.

83 O’Neill, ‘A Case of Jealousy’, 224.

84 Savage, ‘Jealousy’, 722. Also, L. F. Winslow, *Mad Humanity: Its Forms Apparent and Obscure* (London: C. A. Pearson, Ltd., 1898), 156. Some alienists believed Shakespeare accurately described the passions: A. O. Kellogg, *Shakespeare’s Delineations of Insanity, Imbecility and Suicide* (New York: Hurd and Houghton, 1866), 9, 10.

85 Debay quoted in Belenky, *Anxiety*, 28.

86 Conley, *Certain*, 155

87 Wiener, *Men of Blood*, 235; Ruth Harris, *Murders and Madness: Medicine, Law and Society in the Fin de Siècle* (Oxford: Clarendon Press, 1989), 285–320.

88 ‘Jealousy’, *Saturday Review* (March 1863), 298–300.

89 Henry Havelock Ellis, *The Criminal*, 2nd edn (London: Walter Scott Ltd., 1895), 2, 145.

90 Martin Wiener, *Reconstructing the Criminal: Culture, Law and Policy in England, 1830–1914* (Cambridge: Cambridge University Press, 1990), 22.

91 ‘The Murder and Attempted Suicide at Bradford’, *Leicester Chronicle and Leicestershire Mercury*, 8 February 1879; ‘The Woolwich Murder’, *Morning Post*, 6 January.

92 Most patients were able to read and write. See for example, *Reports of the Superintendent and Chaplain of Broadmoor Criminal Lunatic Asylum for the Year 1873* (London: George E. Eyre and William Spottiswoode, 1874), 33; *Reports of the Superintendent and Chaplain of Broadmoor Criminal Lunatic Asylum with Statistical Tables, For the Year 1880* (London: George E. Eyre and William Spottiswoode, 1881), 21; *Reports of the Superintendent and Chaplain of Broadmoor Criminal Lunatic Asylum for the Year 1888* (London: George E. Eyre and William Spottiswoode, 1889), 28. For records recording the ‘imperfect’ level of education amongst ‘jealous’ murderers see for example, D/H14/D2/2/1/963/2, Schedule A; D/H14/D2/2/1/714/1, Schedule A; D/H14/D2/2/1/366/1.

93 ‘The Murder and Attempted Suicide at Bradford’, *Leicester Chronicle*.

94 Alexander Bain, *The Emotions and the Will*, 3rd edn (London: Longman, Green and Co., 1875), 146.

95 ‘The Murder and Attempted Suicide at Bradford’, *Leicester Chronicle*.

96 Martin Wiener, ‘The Sad Story of George Hall: Adultery, Murder and the Politics of Mercy in Mid-Victorian England’, *Social History*, 1999, 24, 175–95, 192.

97
*Old Bailey Proceedings Online* (www.oldbaileyonline.org, version 7.0, 29 October 2012), August 1881, trial of James Hughes (73) (t18810802–725).

98 D/H14/D2/2/1/1212/17, letter to Nicolson.

99 Conley, *Certain*, 113.

100 Lawrence Stone, *Family, Sex and Marriage in England 1500–1800* (London: Weidenfeld and Nicolson, 1977).

101 ‘The Woolwich Murder’, *Morning Post*, 6 January.

102 J. Cuthbert Hadden, *Are You Married? Papers on Love, Courtship, Marriage and Kindred Subjects* (Glasgow: Morison Brothers, 1894), 25.

103 Francoise Barret-Ducrocq, *Love in the Time of Victoria: Sexuality, Class and Gender in Nineteenth-Century London* (London and New York: Verso, 1992), 115, 118.

104 ‘The Shocking Murder at Woolwich’, *Lloyds Weekly Newspaper*, 4 January 1885.

105 D/H14/D2/2/1/1239/6, memorandum; ‘The Woolwich Murder’, *Morning Post*, 6 January; ‘The Woolwich Tragedy’, *Morning Post*, 31 December 1884, 6.

106 ‘The Woolwich Murder’, *Morning Post*, 6 January; ‘The Woolwich Tragedy’, *Morning Post*, 31 December.

107
*Ibid*.

108 D/H14/D2/2/1/1239/3, Schedule A.

109 ‘Criminal Lunatics’, *The Lancet*, 14 March 1884.

110 ‘Insanity and Attempted Murder’, *Standard*, 8 August 1881, 6.

111 ‘Shocking Wife Murder at Wolverhampton’, *Western Mail*, 5 May 1871.

112 ‘Alleged Murder by an Artist’, *Leicester Chronicle and the Leicestershire Mercury*, 30 July 1881; ‘Fearful Murder in York’, *Sheffield and Rotherham Independent*, 10 June 1871, 11.

113 For the history of provocation, Jeremy Horder, *Provocation and Responsibility* (Oxford: Clarendon, 1992), 5–22.

114 ‘Rev. V. Wright. “The Mansfield Murders”’, *Journal of Mental Science*, 1896, 42, 451–6, 454.

115 Horder, *Provocation*, 85; James Fitzjames Stephen, ‘The Definition of Murder’, *London Quarterly Review*, 1866, 26, 428–52, 436.

116 Chadwick, *Bureaucratic Mercy*, 313.

117 ‘The Assize’, *The Times*, 15 November 1884, 11.

118 D/H14/D2/2/1/186/21, letter to Nicolson. This opinion was expressed in other cases. Rowbotham, ‘“Only when drunk”’, in D’Cruze, ed., *Everyday Violence*, 160, 162.

119 Wiener, *Men of Blood*, 201, 205 and Conley, *Certain*, 112, 154.

120 ‘Rev. V. Wright’, 454.

121
*Ibid*., 453–4.

122 Quoted in Mowat, *Morbid Jealousy*, 33.

123 Harris, *Murder and Madness*, 285–92; Stearns, ‘Jealousy in Western History’, 7.

124 Stearns, *Jealousy*, 28.

125 Wiener, *Men of Blood*, 282–3; Roger Smith, *Trial by Medicine: Insanity and Responsibility in Victorian Trials* (Edinburgh: Edinburgh University Press, 1981).

126 ‘The Bradford Murder’, *Leeds Mercury*, 19 November 1878.

127
*Ibid*.

128 For violence and cohabitation, Ginger Frost, ‘He Could Not Hold His Passions: Domestic Violence and Cohabitation in England (1850–1905)’, *Crime, Histoire et Sociétés*, 2008, 12, 25–44.

129 ‘The Murder in Southwark’, *Liverpool Mercury*, 16 April 1866.

130 Old Bailey Proceedings Online (www.oldbaileyonline.org, version 7.2, 14 November 2015), June 1866, trial of DANIEL BELOE (23) (t18660611–575).

131 Discrepancy between lay and medical opinion in the courtroom was not new. Joel Peter Eigen, ‘Lesion of the Will: Medical Resolve and Criminal Responsibility in Victorian Criminal Trials’, *Law & Society Review*, 1999, 33, 425–59.

132
*OBPO*, Beloe’s trial.

133
*Ibid*.

134 John Charles Bucknill, ‘Trial of Robert Handcock for the Murder of his Wife, Phillipa Handcock, at the Devon Winter Assizes, before Mr. Baron Parke, Dec. 10^th^, 1855. Plea of Insanity’, *Journal of Mental Science*, 1856, 2, 245–53, 245.

135 Eigen, ‘Diagnosing Homicidal Mania”, 433–56; Roger Smith, ‘Defining Murder and Madness: An Introduction to Medicolegal Belief in the Case of Mary Ann Brough’, *Knowledge and Society*, 4 (1982), 173–225; Roger Smith, ‘The Boundary Between Insanity and Criminal Responsibility in Nineteenth-Century England’, in Andrew Scull, ed., *Madhouses, Mad-Doctors, and Madmen: The Social History of Psychiatry in the Victorian Era* (London: The Athlone Press, 1981), 363–77, 363.

136 Conley, *Certain*, 113.

137 My research suggests that lawyers referred to jealousy more frequently in court after 1870, when increased discussion of jealousy as both a cause and a symptom of insanity in medical literature may have boosted confidence that claims of jealousy could obtain an insanity verdict or a reduced sentence. A search of OBPO returns no murder trials in which ‘jealousy’ was recorded anywhere in the proceedings where insanity was the verdict between 1864 and 1885. Between 1864 and 1900 jealousy appeared in the proceedings of nineteen men’s murder trials; twelve of these after 1880.

138 ‘Horrible Murder of a Wife at York’, *York Herald*, 10 June 1871, 9; ‘Fearful Murder in York’, *Sheffield and Rotherham Independent*, 10 June 1871, 11.

139 ‘The Shocking Wife Murder at York’, *Illustrated Police News*, August 1871. Cook’s trial was the first time intemperance was successfully used as an insanity defence: Wiener, *Men of Blood*, 279

140 George Fielding Blandford, ‘The Lumleian Lectures on the Diagnosis, Prognosis, and Prophylaxis of Insanity’, *BMJ*, 6 April 1895, 741–5, 741.

141 A. Ben-Ze’ev, ‘Jealousy and Romantic Love’, in Sybil. L. Hart and Maria Legerstee eds, *Handbook of Jealousy: Theory, Research, and Multidisciplinary Approaches* (Chichester: Wiley-Blackwell, 2010), 40–54, 42.

142 Millingen, *Mind*, 384.

143 D/H14/D2/2/1/1256, Cantrill’s case file; D/H14/D2/2/1/1258/3, Willoughby’s case file.

144 D/H14/D2/2/1/963/7, medical report.

145 D/H14/D2/2/1/963/11, ‘The Case of Anthony Owston’, *Bradford Observer.*

146 D/H14/D2/2/1/1067/4, letter to Orange.

147 D/H14/D2/2/1/1258/12, medical report.

148 D/H14/D2/2/1/1239/39–51, press cuttings.

149 D/H14/D2/2/1/1239/38, medical report.

150 D/H14/D2/2/1/1239/6–8, memorandum.

151 Roger Smith, *Inhibition: History and Meaning in the Sciences of Mind and Brain* (London: Free Association Books, 1992), 163.

152 James Taylor, ‘Jackson, John Hughlings (1835–1911)’, rev. Walton of Detchant, *Oxford Dictionary of National Biography*, Oxford University Press, 2004, <http://0-www.oxforddnb.com.catalogue.ulrls.lon.ac.uk/view/article/34137>, accessed 13 May 2013.

153 D/H14/D2/2/1/1239/38, medical report.

154 David Nicolson, ‘The Morbid Psychology of Criminals’, *Journal of Mental Science*, 21 (April 1875), 18–31, 18–19.

155 See the following case files: D/H14/D2/2/1/186; D/H14/D2/2/1/714; D/H14/D2/2/1/963; D/H14/D2/2/1/1013.

156 D/H14/A2/1/1/7, Annual Report (1881), 55.

157 Andrew Scull, *The Most Solitary of Afflictions: Madness and Society in Britain, 1700–1900* (New Haven, CT: Yale University Press, 1993), 236.

158 Akihito Suzuki, ‘Framing Psychiatric Subjectivity: Doctor, Patient and Record-Keeping and Bethlem in the Nineteenth Century,’ in Joseph Melling and Bill Forsythe, eds, *Insanity, Institutions and Society, 1800–1914: A Social History of Madness in Comparative Perspective* (London and New York: Routledge, 1999), 115–36, 116; Katherine Hunter, *Doctors’ Stories: The Narrative Structure of Medical Knowledge* (Princeton, NJ: Princeton University Press, 1991), 13.

159 Jane Wood shows that cultural assumptions about gender and illness crossed national boundaries. Wood, *Passion*, 19.

160 Gesa Stedman, *Stemming the Torrent: Expression and Control in the Victorian Discourses on Emotion, 1830–1872* (Aldershot: Ashgate, 2002), 185.

